# Altered paracellular cation permeability due to a rare *CLDN10B* variant causes anhidrosis and kidney damage

**DOI:** 10.1371/journal.pgen.1006897

**Published:** 2017-07-07

**Authors:** Joakim Klar, Jörg Piontek, Susanne Milatz, Muhammad Tariq, Muhammad Jameel, Tilman Breiderhoff, Jens Schuster, Ambrin Fatima, Maria Asif, Muhammad Sher, Katrin Mäbert, Anja Fromm, Shahid M. Baig, Dorothee Günzel, Niklas Dahl

**Affiliations:** 1 Department of Immunology, Genetics and Pathology, Science for Life Laboratory, Uppsala University, Uppsala, Sweden; 2 Institute of Clinical Physiology, Charité–Universitätsmedizin Berlin, Charité, Berlin, Germany; 3 Human Molecular Genetics Laboratory, National Institute for Biotechnology and Genetic Engineering (NIBGE), PIEAS, Faisalabad, Pakistan; 4 Department of Pediatric Nephrology, Charité–Universitätsmedizin Berlin, Berlin, Germany; Columbia University, UNITED STATES

## Abstract

Claudins constitute the major component of tight junctions and regulate paracellular permeability of epithelia. Claudin-10 occurs in two major isoforms that form paracellular channels with ion selectivity. We report on two families segregating an autosomal recessive disorder characterized by generalized anhidrosis, severe heat intolerance and mild kidney failure. All affected individuals carry a rare homozygous missense mutation c.144C>G, p.(N48K) specific for the claudin-10b isoform. Immunostaining of sweat glands from patients suggested that the disease is associated with reduced levels of claudin-10b in the plasma membranes and in canaliculi of the secretory portion. Expression of claudin-10b N48K in a 3D cell model of sweat secretion indicated perturbed paracellular Na^+^ transport. Analysis of paracellular permeability revealed that claudin-10b N48K maintained cation over anion selectivity but with a reduced general ion conductance. Furthermore, freeze fracture electron microscopy showed that claudin-10b N48K was associated with impaired tight junction strand formation and altered cis-oligomer formation. These data suggest that claudin-10b N48K causes anhidrosis and our findings are consistent with a combined effect from perturbed TJ function and increased degradation of claudin-10b N48K in the sweat glands. Furthermore, affected individuals present with Mg^2+^ retention, secondary hyperparathyroidism and mild kidney failure that suggest a disturbed reabsorption of cations in the kidneys. These renal-derived features recapitulate several phenotypic aspects detected in mice with kidney specific loss of both claudin-10 isoforms. Our study adds to the spectrum of phenotypes caused by tight junction proteins and demonstrates a pivotal role for claudin-10b in maintaining paracellular Na^+^ permeability for sweat production and kidney function.

## Author summary

Claudins are tight junction proteins forming paracellular barriers that are critical for normal development and homeostasis. The tissue specific paracellular barrier properties are determined by the protein composition of tight junctions that regulates the permeability of solutes and water between different compartments of the body. We show, for the first time, that a mutation in claudin-10b, forming paracellular cation channels in different tissues, causes perturbed Na^+^ selectivity through altered tight junction formation and function as well as increased degradation of the protein. The mutation is associated with the inability to sweat (anhidrosis) and heat intolerance as well as abnormal cation reabsorption, hypermagnesemia and kidney damage. Our combined findings show that the claudin-10b-mediated paracellular Na^+^ transport is required for normal sweat production and for the regulation of cation homeostasis in the kidneys.

## Introduction

Epithelial and endothelial cells constitute sheets that divide organs into functional compartments. Homeostasis of different organ and body compartments are dependent on epithelial cells and their paracellular barrier that prevents solutes and water from leaking between the cells. The tissue specific paracellular barrier properties are determined by the protein composition of tight junctions (TJs) [1]. Some TJs form truly impermeable barriers whereas others contain paracellular channels for selective exchange of small ions between compartments. The selectivity is largely determined by the expression of specific members of the claudin protein family [2, 3]. In mammals, the claudin protein family comprises 27 members consisting of small, highly conserved transmembrane proteins with four transmembrane helices and two extracellular loops [2]. The first extracellular loop contributes to ion selectivity in channel-forming claudins [4] and the second extracellular loop is of importance for claudin-claudin interactions [5, 6]. The critical roles for claudin proteins in development and homeostasis are documented by mouse models as well as by some rare human diseases. Early lethality or specific phenotypes have been identified in mice targeted for the genes encoding claudin 1, 2, 4, 5, 7, 10, 11, 15, 16 18, and 19, respectively [7]. In humans, variants in the *CLDN16* and *CLDN19* genes are associated with hypomagnesemia, hypercalciuria and nephrocalcinosis (OMIM #248250 and OMIM #248190), *CLDN14* mutations causes a form of autosomal recessive deafness (OMIM #614035) and *CLDN1* mutations have been described in rare patients with ichthyosis, leukocyte vacuoles, alopecia, and sclerosing cholangitis (ILVASC; OMIM#607626) [8–12].

*CLDN10* encodes two distinct isoforms that differ in their amino terminal transmembrane helix and the first extracellular loop [13]. Expression of isoform 10a is restricted to the kidney and uterus while isoform 10b is ubiquitously expressed. Claudin-10a forms an anion-selective channel whereas claudin-10b forms a water-impermeable cation-selective channel with preference for Na^+^ [13, 14]. In mice, loss of claudin-10b in the distal segments of the nephron has been shown to cause impaired Na^+^ permeability as well as increased Ca^2+^ and Mg^2+^ resorption that lead to hypermagnesemia and nephrocalcinosis [15]. However, the effect of impaired claudin-10 function in humans has remained unknown.

Here, we report on a homozygous *CLDN10b* variant c.144C>G, p.(N48K), in 13 individuals from two kindreds presenting with anhidrosis, alacrima (inability to produce tears), xerostomia (dry mouth) and kidney failure associated with hypermagnesemia. We demonstrate that the claudin-10b N48K variant has pathogenic consequences, since it alters paracellular Na^+^ transport in a model for sweat secretion, claudin oligomerization tight junction formation at cell-cell contacts, electrophysiological properties of epithelial monolayers and the amount of claudin-10b in the cell membrane. Together, our data reveal mechanisms caused by impaired claudin-10b function and its phenotypic consequences.

## Results

### Phenotypic features of the study subjects

**Fig 1:**
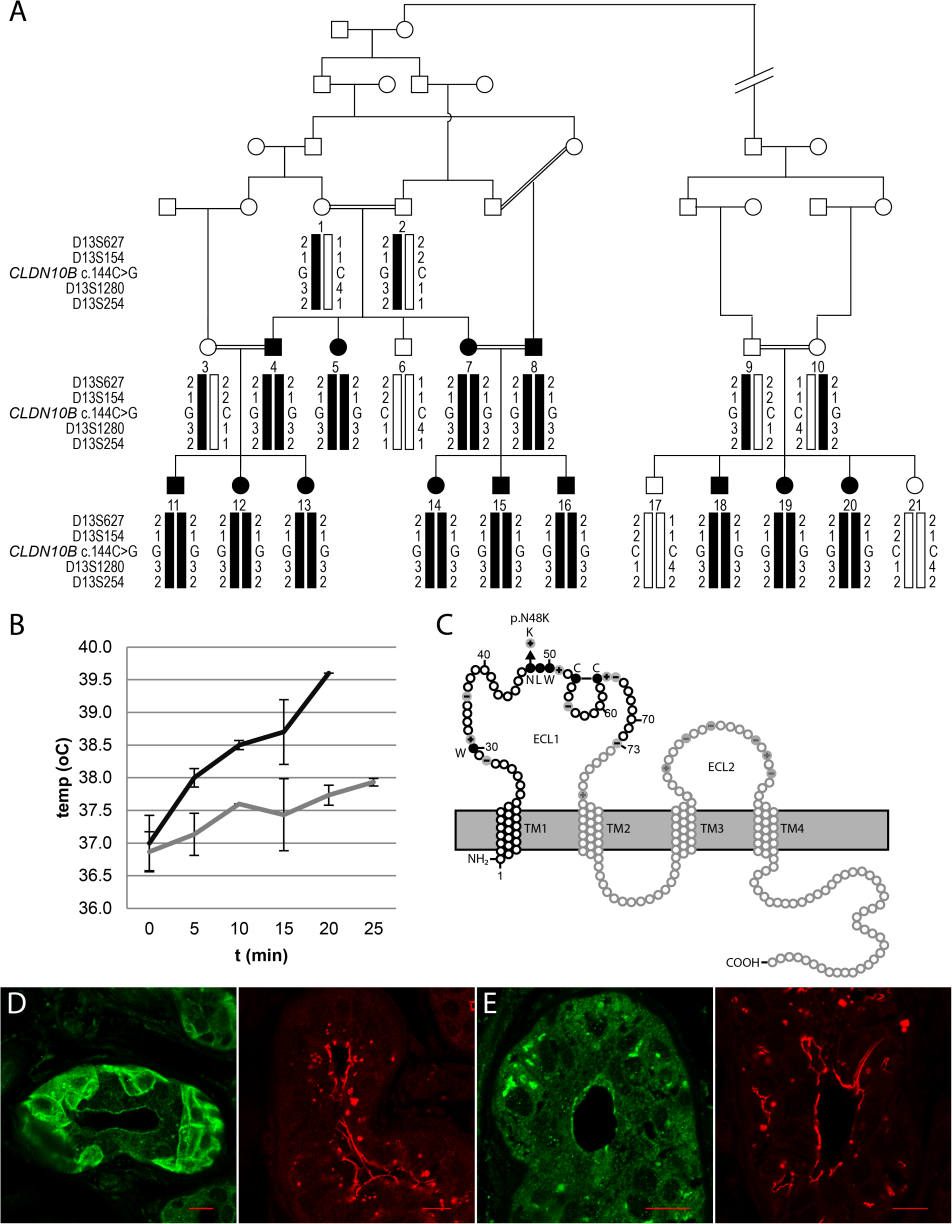
The *CLDN10b* specific gene variant segregates anhidrosis and mild kidney failure. (A) Pedigree of the consanguineous Pakistani family segregating anhidrosis and heat intolerance, alacrima, xerostomia, mild kidney failure and Mg^2+^ retention (black filled symbols). All affected individuals are homozygous for chromosome 13q32 marker alleles flanking the *CLDN10* gene with the missense variant c.144C>G (black bars). (B) Body temperature measurements at rest over 25 min when exposed to 45°C and 45% humidity in affected (n = 2; black line) and age-matched controls (n = 3; grey line). The patients interrupted the study after 20 min due to distress. (C) The N48K variant is located within the 1^st^ extracellular loop (ECL1) and results in the introduction of the positively charged Lysine in the claudin consensus sequence W- G/NLW-C-C (filled amino acids). The first 73 amino acids (black circles) are specific for the claudin-10b isoform encoded by exon 1b. Grey circles correspond to amino acids shared with the claudin-10a isoform. +/- indicate charged residues in the extracellular loops. ECL–extracellular loop. TM–Transmembrane segment. (D-E) Immunostaining of the secretory portion of sweat glands from a healthy control (D) and from the affected ind. 15 (E). In the control, claudin-10 (green) is predominantly found in the canaliculi, the cell peripheries and in membranes lining the lumen. Strong occludin staining (red) is found lining the main lumen of the sweat gland. In the patient, claudin-10 signal (green) is observed in membranes facing the lumen but shows a more even intracellular distribution without accumulation in canaliculi but in spots resembling vesicles. A strong occludin signal (red) is observed both in the canaliculi and in membranes lining the main lumen. Scale bars: 20μm.

Two distantly related Pakistani kindreds segregating heat intolerance and generalized anhidrosis from birth were identified. Altogether 13 affected individuals were ascertained and several loops of consanguinity suggested an autosomal recessive mode of inheritance (Fig 1A). The anhidrosis was associated with inability to produce tears (alacrima) and dry mouth (xerostomia) in all 13 individuals. In addition, several affected members suffered from recurrent kidney pain due to nephrolithiasis with onset in adolescence (individuals 4, 7, 12 and 14). Heat intolerance was assessed by exposure to heat during 20 minutes (ind. 4 and ind. 11) and resulted in a rapidly increased body temperature from 37°C to 39.6°C when compared to gender- and age-matched control individuals (Fig 1B). The increased skin temperature was accompanied with an increase in heart rate from 106 bpm to 170 bpm. Perspiration was sparse or absent in patients when using the starch-iod test applied on different body parts consistent with generalized anhidrosis (S1 Fig).

Analysis of serum electrolytes revealed increased Mg^2+^ levels but no other overt abnormalities (Table 1). An abnormal renal reabsorption of cations was reflected in urine spot samples that showed low concentrations of Mg^2+^ as well as Ca^2+^ in six affected individuals. Parathyroid hormone (PTH) was analyzed in two affected individuals and revealed a two- and three-fold increase, respectively, when compared to normal levels. The same two individuals had reduced 25-hydroxy vitamin D levels. In combination, these observations suggested secondary hyperparathyroidism and kidney damage and further supported by an eGFR in the lower normal range (Table 1). In contrast, creatinine, urea and bicarbonate in serum were normal (n = 4) as well as the 24h urine production (n = 2; 1400ml and 1450ml, respectively). Computer tomography (CT) scans of kidneys (n = 2) were normal. Pancreatic function was assessed by analysis of amylase and lipase levels that turned out normal (n = 2). Lung functions were investigated by spirometry and turned out normal (n = 2; S1 Table).

**Table 1:** Results from urine (spot) and serum analysis in six affected family members.

Case* Age (years)	4 47†	11 27	12 25	13 23	15 27	16 35	Average	Patient ranges (95% CI)	Normal ranges	
***URINE (spot)***										
**Ph**	na	na	5	5	5	na	5	5	4.5–8.0	
**Osmolality**	na	406	401	338	401	438	397	356–438	300–1200	mOsmol/kg
**Urea**	na	na	*399*	*253*	*204*	na	*285*	170–400**	*847–2967*	*mg/dl*
**Mg**^**2+**^	*2*.*9*	*6*.*7*	*4*.*9*	*3*.*0*	*4*.*1*	na	*4*.*3*	*2*.*9–5*.7**	*7*.*3–12*.*2*	*mg/dl*
**K**^**+**^	26	33	30	16	19	34	27	20–34	20–80	mmol/l
**Ca**^**2+**^	*0*.*5*	*0*.*2*	*0*.*3*	*0*.*1*	*0*.*8*	na	*0*.*4*	*0*.*1–0*.6**	*6*.*8–21*.*3*	*mg/dl*
**Na**^**+**^	103	107	98	110	145	na	113	96–129	54–150	mmol/l
**Cl**^**-**^	119	110	109	109	147	na	119	104–133	46–168	mmol/l
***SERUM***										
**Ca**^**2+**^	na	9	na	na	na	9	9	9	8.4–10.2	mg/dl
**Mg**^**2+**^	*3*.*0*	*2*.*7*	*2*.*6*	*2*.*5*	*3*.*0*	*2*.*8*	*2*.*8*	*2*.*6–2*.9**	*1*.*5–2*.*5*	*mg/dl*
**Na**^**+**^	143	140	140	138	139	139	140	138–142	133–145	mmol/L
**K**^**+**^	4.7	3.6	4.6	5.1	5.1	4.5	4.6	4.1–5.1	3.3–5.1	mmol/l
**Cl**^**-**^	107	101	99	94	95	95	99	94–103	95–108	mmol/l
**Phosphorus**	na	na	3.1	4.3	4.1	4.6	4.0	3.4–4.7	2.7–4.5	mmol/l
**Urea**	na	18	13	14	12	14	14	11–17	6–20	mg/dl
**Creatinine**	na	na	0.7	0.6	0.7	na	0.7	0.6–0.7	0.5–0.9	mg/dl
**PTH**	na	*184*	na	na	na	*117*	*151*	97–204**	*11–67*	*pg/ml*
**25OHVitD**	na	36	na	na	na	12	24	5–43	40–100	ng/ml
**Bicarbonate**	na	23	na	na	na	23	23	23	22–29	mmol/l
**eGFR**	na	74	na	na	na	72	73	71–74	>60	ml/min/1.79
**Amylase**	na	41	na	na	na	69	55	33–77	30–110	U/l
**Lipase**	na	21	na	na	na	49	35	12–57	<60	U/l

*: Numbers of cases are indicated in pedigree (Fig 1A).

**: 95% confidence interval (CI)

for patient values outside normal reference range. na: data not available.

†: age of death. Normal ranges are derived from populations of mixed ethnicities.

### Identification of the *CLDN10b* missense variant c.144C>G, p(N48K) associated with anhidrosis and kidney damage

Autozygosity mapping of the family identified a homozygous region of 235 consecutive SNPs spanning a 2 Mb region on chromosome 13q32. Fine mapping using microsatellite markers confirmed homozygosity and linkage analysis resulted in a maximum two-point logarithm of odds (LOD) score of 4.25 (Fig 1A). We enriched genomic DNA spanning the candidate region on chromosome 13q32 (average fold enrichment x386) from one affected family member. Variant detection using v2.1 of the LifeScope Software (Life Technologies) revealed only two homozygous missense variants in the linked homozygous region: A c.144C>G, p.(N48K) in *CLDN10b* (NM_006984.4) and a c.982T>G, p.(S328A) in *UGGT2* (NM_020121.3), respectively. The c.982T>G variant in *UGGT2* was annotated as a SNP in dbSNP132 (rs816142, mean allele frequency 0.14; ExAC) and without predicted severe impact on function. The *CLDN10b* gene variant c.144C>G results in the change of an uncharged asparagine into a positively charged lysine at amino acid position 48 in the first extracellular loop of the protein (Fig 1C)**.** The N48 residue is part of the conserved claudin consensus motif (W-G/NLW-C-C) and the substitution was predicted to have a severe impact on claudin-10b function. The c.144C>G transition was found in a homozygous state in all affected family members and in a heterozygous state in unaffected parents. Furthermore, the variant was excluded on 600 control chromosomes from Pakistan and it was not present in the ExAC database (http://exac.broadinstitute.org/) suggesting this variant to be very rare [16].

### Localization of claudin-10b and other tight junction proteins in sweat glands

The finding of a homozygous missense variant in the *CLDN10b* gene suggested altered paracellular ion permeability as a mechanism of disease. Anhidrosis was an early and prominent symptom of the disease and hence we performed histological investigations of sweat glands in forearm punch biopsies of two affected individuals. The morphology and number of sweat glands appeared normal and immunohistochemical analysis of claudin-10b, the single claudin-10 isoform expressed in sweat glands, revealed strong signals in cells of the secretory portions without visible differences between patients and healthy controls. Immunofluorescence staining of sweat glands revealed that claudin-10b is localized in the periphery of cells from the secretory portions (corresponding to the clear cells), most likely in the basal membrane infoldings, as well as in the plasma membranes lining the canaliculi and, to a lesser extent, the lumen. Claudin-10b staining co-localized with that of the “sealing” claudin-1 and claudin-3 (S2A and S2B Fig). Furthermore, co-staining of claudin-10b and the TJ protein occludin confirmed an overlap in membranes lining the canaliculi and the lumen. In addition, occludin co-localized with the TJ protein ZO1 in both canalicular and luminal membranes (S2C and S2D Fig). Compared to healthy subjects, the claudin-10b staining in sweat glands of two affected individuals (ind. 4 and ind. 15) showed staining in membranes facing the lumen, but was otherwise mainly intracellular and dramatically reduced in the canaliculi (Fig 1D and 1E). The pattern suggested impaired association of claudin-10b to TJ and an intracellular accumulation, possibly as degraded products, in vesicles. In contrast, immunofluorescence staining of the TJ protein occludin did not reveal any differences in distribution when comparing sweat glands from patient to those of healthy controls (Fig 1D and 1E).

### Claudin-10b induced lumen expansion of MDCK-C7 cysts is reduced by claudin-10b N48K

**Fig 2:**
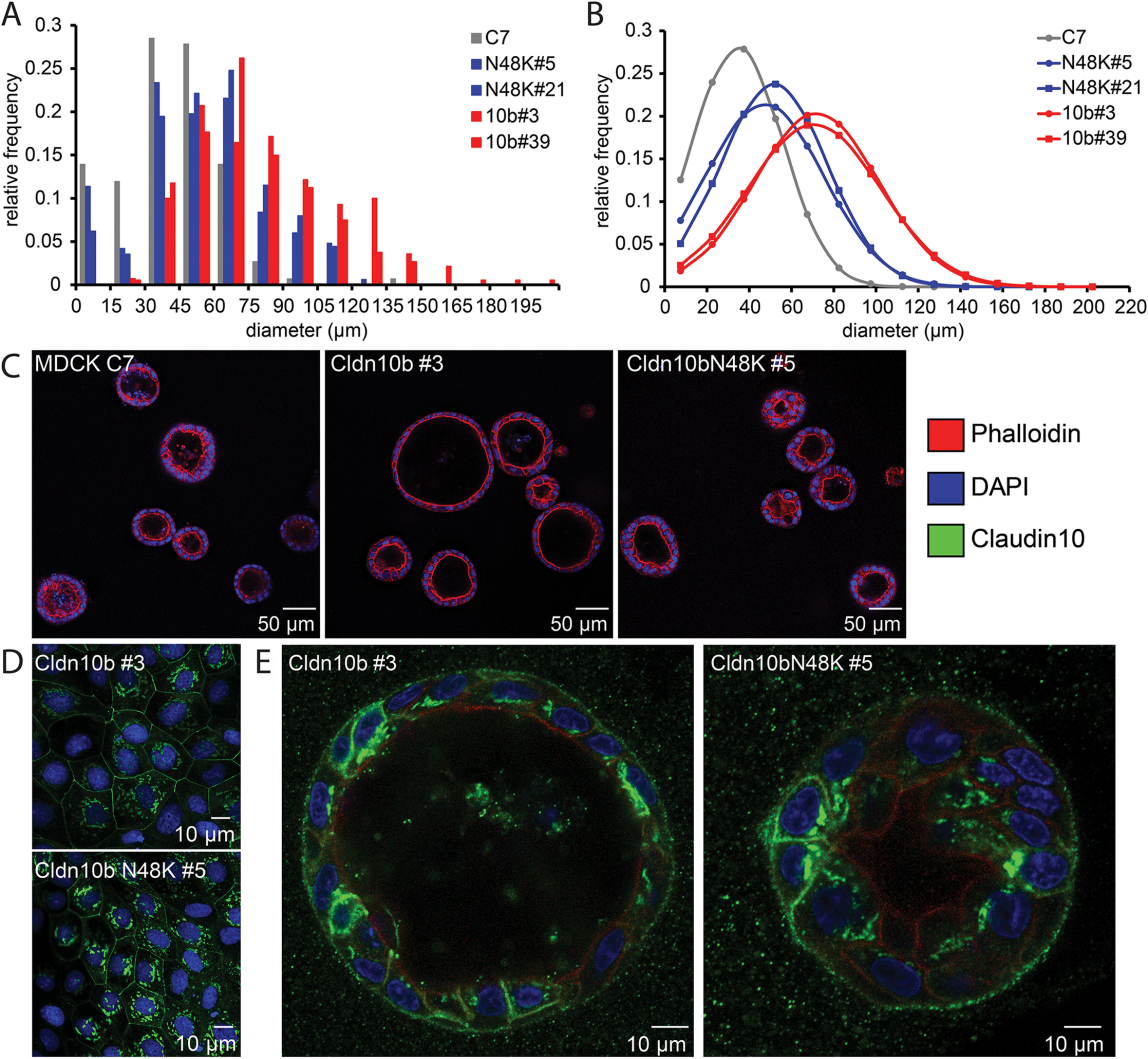
Reduced size of cysts formed by MDCK-C7 cells transfected by claudin-10b N48K. (A) Histogram of the size distribution of cyst lumen diameter (grey, untransfected control, n = 151 cysts [10 different z-stacks]; red, claudin-10b WT transfected cells: clone #3, n = 140 [9], clone #39, n = 187 [11]; blue claudin-10b N48K transfected cells: clone #5, n = 167 [9]; clone # 21, n = 113 [7]). (B) Normal distribution of the data shown in (A) to visualize the shift in lumen size in the presence of claudin-10b. (C) Representative images of cysts (red, actin-staining by phalloidin-Alexa 594; blue, nuclei stained with DAPI), (D) TJ localization of N48K and WT claudin-10b in 2D culture. (E) Presence of WT and N48K claudin-10b (green) in 3D culture (co-staining as in D).

To mimic sweat secretion in a model system we cultured MDCK-C7 cells in Matrigel. Under these conditions, MDCK-C7 cells form three-dimensional cysts (apical side towards the lumen). As demonstrated by Bagnat et al. 2007, lumen formation and expansion in MDCK cells depends on transcellular Cl^-^ secretion that is accompanied by paracellular Na^+^ movement. The resulting osmotic gradient drives water into the cyst lumen. Cyst lumen diameters were shown to increase, when cells were transfected with the cation channel-forming zebrafish claudin-15 [17]. We therefore hypothesized that the presence of the cation channel forming claudin-10b should similarly enhance fluid secretion. As shown in Fig 2A–2C, this is indeed the case: claudin-10b expression in MDCK-C7 cells resulted in an increase in the mean lumen diameter (untransfected control, 33.5 ± 2.6 μm, n = 10 different z-stacks [total of 151 cysts]; claudin-10b WT clone #3, 70.7 ± 4.7 μm, n = 9 [140], p < 0.01; claudin-10b WT clone #39, 70.6 ± 5.6 μm, n = 11 [187], p < 0.01, student’s t-test with Bonferroni-Holm correction, mean ± SEM). Claudin-10b N48K transfected MDCK-C7 cells on the other hand, formed cysts that showed considerably less lumen expansion (claudin-10b N48K clone #21, 52.68 ± 1.7 μm, n = 7[113], p < 0.01 vs control, p < 0.01 vs WT claudin-10b #39; claudin-10b N48K clone #5, 48.5 ± 4.4 μm, n = 9[167], p < 0.05 vs control, p < 0.01 vs WT claudin-10b #3). Both WT and N48K claudin-10b resided in the TJ in 2D as well as 3D cultures (Fig 2D and 2E), however, the mutated variant showed a distribution that suggested an increased intracellular accumulation (Fig 2D).

### Claudin-10b N48K affects relative permeability P_Na_/P_Cl_ and show increased tendency for degradation

**Fig 3:**
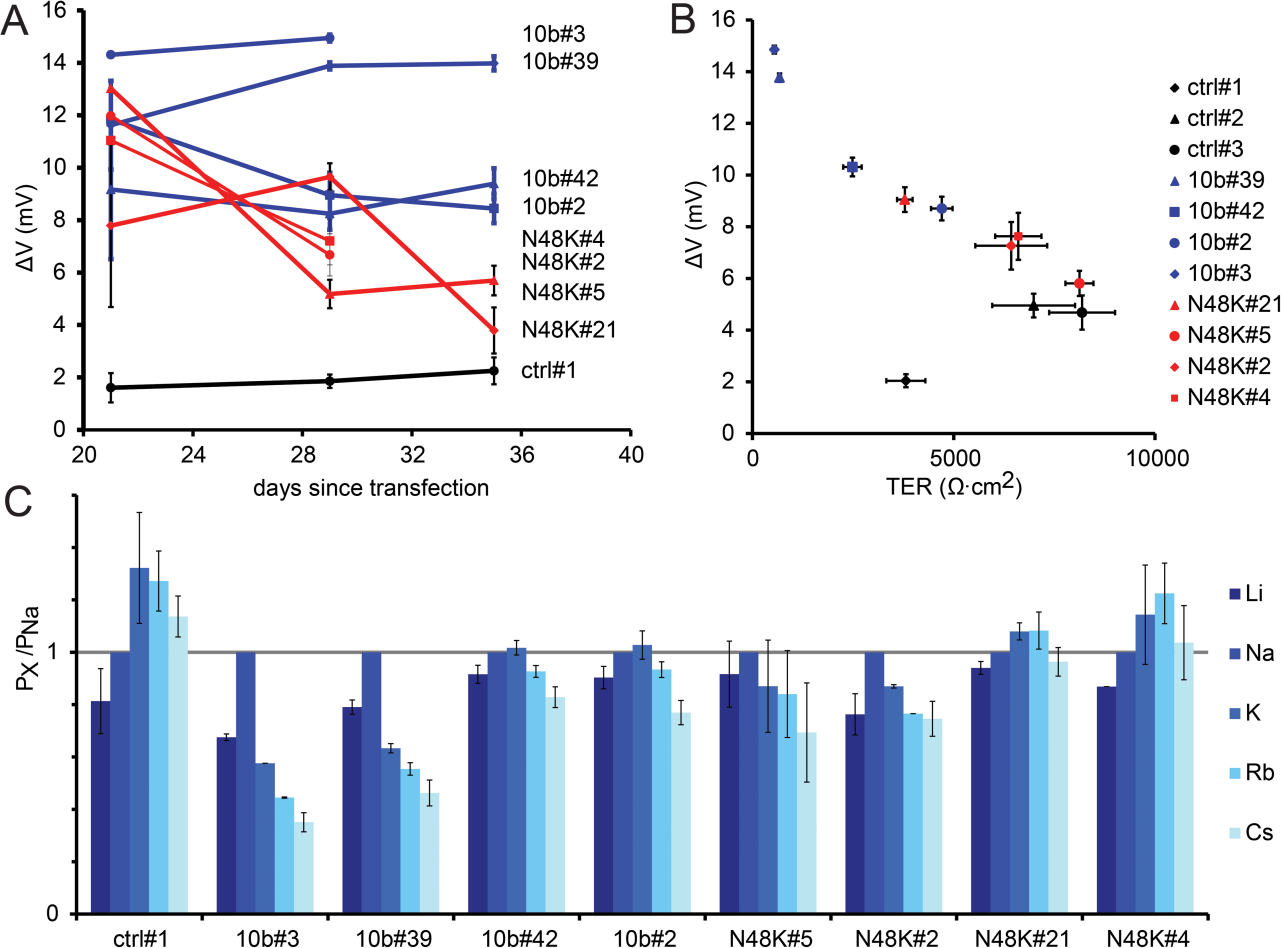
Claudin-10b N48K alters electrophysiological properties in MDCK-C7 cell layers. (A) Dilution potentials (and thus the permeability ratio for Na^+^ and Cl^-^; P_Na_/P_Cl_) of cell layers expressing claudin-10b WT (blue) and claudin-10b N48K (red) are significantly higher than those of control cell layers (black; empty vector). Cell layers transfected with claudin-10b WT maintain these properties over several weeks whereas cell layers transfected with claudin-10b N48K lose these properties over a time-course of three weeks. n = 1–28, means +/- SEM. (B) Expression of claudin-10b WT (blue symbols) cause reduced TER (x-axis) and increased dilution potentials (y-axis) compared to vector transfected controls (black symbols). The effects when expressing claudin-10b N48K (red symbols) were less pronounced. n = 8–40, means +/- SEM. (C) Permeability for monovalent cations (P_X_) relative to P_Na_. Expression of claudin-10b WT altered Eisenman-sequence for monovalent cations from Eisenman-sequence IV in control layers to Eisenman-sequence VIII to X, depending on transfection strength. Expression of claudin-10b N48K resulted in Eisenman-sequences between III and X. n = 2–13, means +/- SEM.

Claudin-10b acts as a paracellular cation channel that is expressed in multiple tissues. To determine a possible effect of the claudin-10b N48K variant on ion permeability we generated MDCK-C7 cells stably expressing the mutated and the WT proteins. Starting 21 days after transfection we measured dilution potentials at weekly intervals. Initially, the dilution potentials, and thus the ratio for Na^+^ and Cl^-^ permeabilities (P_Na_/P_Cl_), were similar for cell layers expressing claudin-10b WT and claudin-10b N48K, but considerably increased when compared to control cell layers containing an empty vector. However, whereas dilution potentials of claudin-10b WT transfected cell layers remained stable over several passages, dilution potentials of cell layers expressing claudin-10b N48K progressively decreased (Fig 3A), signifying a time-dependent reduction in permeability ratio P_Na_/P_Cl_. Transepithelial resistance (TER) was considerably reduced in claudin-10b WT expressing cell layers, as expected for a channel-containing tight junction (Fig 3B). In contrast, the TER reduction in cell layers expressing claudin-10b N48K was less pronounced and similar to cell layers with a weak expression of claudin-10b WT. Relative permeabilities for the monovalent cations Li, Na, K, Rb and Cs in claudin-10b N48K-expressing cell layers followed Eisenman sequences, resembling cell layers with weak expression of claudin-10b WT (Fig 3C) [14]. These observations strongly suggest a reduced selectivity for Na^+^ over other cations mediated by the p.N48K variant, or a reduced amount of claudin-10b within the tight junction.

To clarify if increased degradation of claudin-10b N48K may contribute to the reduced Na^+^ selectivity, we analyzed the turn-over of the protein in HEK293 cells stably expressing claudin-10b fused to CFP or YFP. For claudin-10b N48K, a higher proportion of cells showed cytosolic CFP/YFP fluorescence, when compared to that of claudin-10b WT (S3A and S3B Fig). This indicates that presence of p.N48K increases cleavage of the CFP/YFP moiety from the fusion protein. Furthermore, Western blot analyses of cell lysates revealed more cleaved products for claudin-10b N48K when compared to claudin-10b WT (S3C and S3D Fig). These data suggest that p.N48K enhances the degradation of claudin-10b in addition to the effect on Na^+^ selectivity.

### The claudin-10b N48K mutation perturbs formation and ultrastructure of tight junction strands

**Fig 4:**
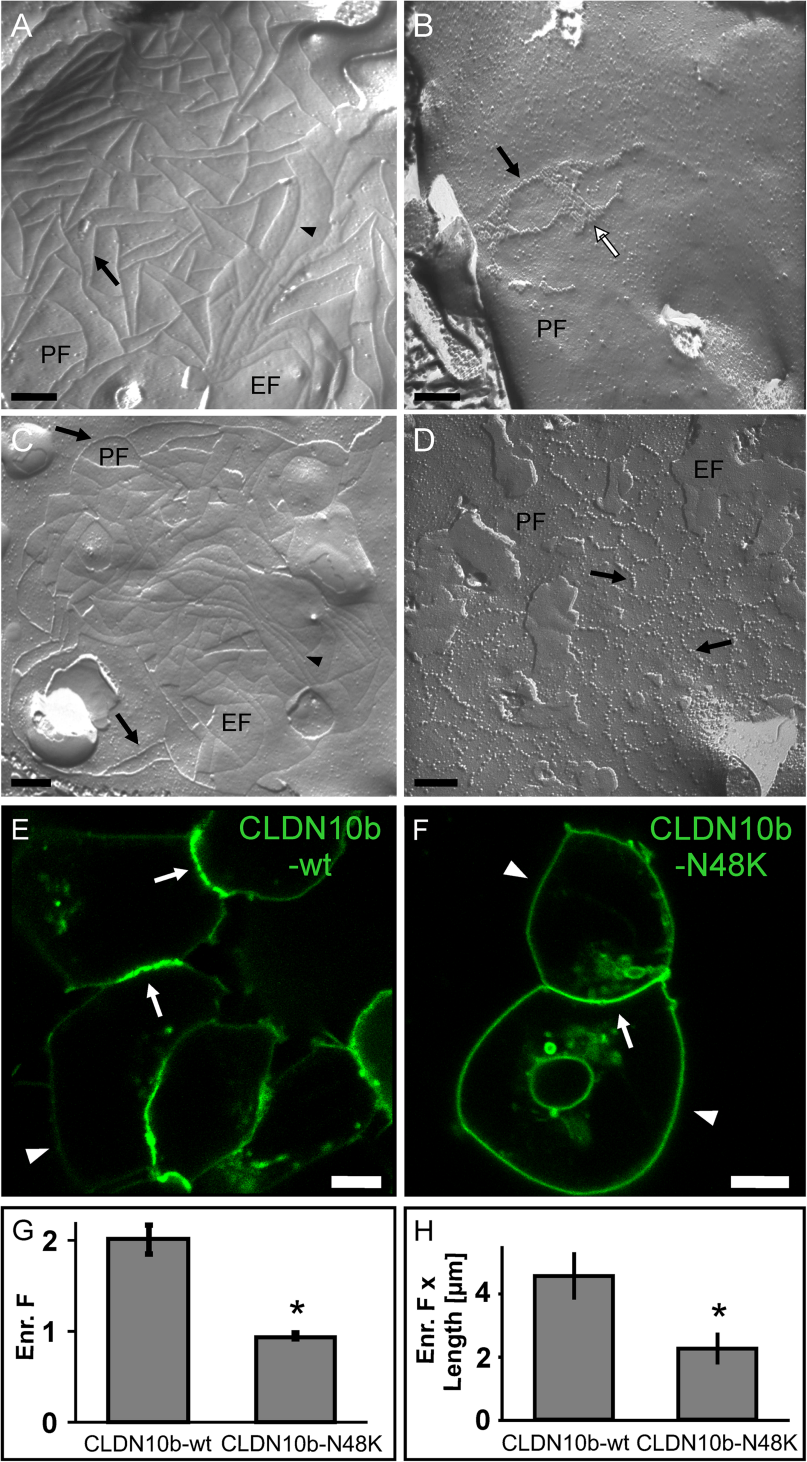
Perturbed formation of tight junctions and inhibition of claudin-10b trans-interactions by the N48K mutation. (A-D) Freeze fracture electron microscopy of HEK293 cells expressing YFP-claudin-10b. (A) Stable expression of wt claudin-10b led to formation of complex mesh-works of branched tight junction strands. Numerous continuous strands (arrow) were detected on the protoplasmic face (P-face, PF) and as particle-free grooves (arrowhead) on the exoplasmic face (E-face, EF) of the plasma membrane. (B) Stable expression of claudin-10b N48K resulted in few tight junction strands and less complex meshworks. Strands were detected on the P-face as rows of rather separated intramembranous particles (black arrow) and two- dimensional particle arrays (white arrow). (C) Similar to the stable expression, transient expression of wt claudin-10b led to mesh-works of continuous tight junction strands detected on the P-face (arrow) and E-face (arrowhead). (D) For transient expression of claudin-10b N48K, tight junction mesh-works were infrequent. Particle-type strands were detected as noncontinuous rows of separated, beaded intramembranous particles (black arrow) on the P-face. Scale bars, 200 nm. (E-H) Inhibition of YFP-claudin-10b trans-interactions by the p.N48K mutation. (E) Transiently expressed claudin-10b WT (green) is enriched at contacts (arrows) between claudin-expressing HEK293 cells suggesting trans-interaction. Arrowheads indicate plasma membrane outside of contacts between two claudin-expressing cells. (F) Transient expression of claudin-10b N48K (green) in HEK293 cells does not show strong enrichment at contacts (arrows) between cells suggesting lack of trans-interaction. (G) For transient expression in HEK293 cells, the enrichment factor (Enr. F) was significantly lower for claudin-10b N48K compared to claudin-10b WT. n = 27–44; *, p < 0.01. (H) For stable expression in HEK293 cells, the product of enrichment factor and length of enrichment (Enr. F x Length) was significantly lower for claudin-10b N48K compared to claudin-10b WT. n = 27–42; *, p < 0.01. Means +/- SEM. Size bars: 5μm.

Since claudin-10b is a TJ protein we sought to further investigate the effect of the p.N48K mutation on claudin-10b-mediated formation of TJs despite apparently normal immunofluorescence staining of occludin in sweat glands of patients. To this end, we expressed claudin-10b in HEK293 cells without endogenous TJs [5]. Formation and reconstitution of TJs after transfection with either YFP-claudin-10b WT or N48K, respectively, were analyzed by freeze fracture electron microscopy (EM). Stable expression of YFP-claudin-10b WT resulted in the formation of typical epithelial TJs with complex meshwork of continuous and branched TJ strands (Fig 4A). Smooth strands with continuity of intramembranous particles were detected on the protoplasmic fracture face (P-face) of the plasma membrane. In contrast, stable expression of YFP-claudin-10b N48K resulted in very few TJ strands and meshwork with a much lower complexity (Fig 4B). In addition, the strands consisted of separated intramembranous particles and partly two-dimensional particle arrays were observed. After transient expression, similar results were obtained as for stable expression: Transfection of YFP-claudin-10b WT resulted in extended meshwork of continuous TJ strands (Fig 4C), whereas expression of YFP-claudin-10b N48K gave rise to a sparser TJ meshwork with discontinuous and beaded intramembranous particles (particle-type strands) on the P-face (Fig 4D). These data suggest that p.N48K inhibits the formation of continuous-type TJ strands by claudin-10b.

To corroborate our findings, we analyzed the capability of claudin-10b for homophilic trans-interaction. We performed a cellular contact enrichment assay in which trans-interaction of claudins is measured from the selective enrichment of the construct of interest at contacts between two claudin-expressing cells [5]. We observed that both YFP-claudin-10b WT and N48K localized primarily to the plasma membrane of HEK293 cells (Fig 4E and 4F). Cells transiently expressing YFP-claudin-10b WT showed a strong contact enrichment indicating trans-interactions whereas cells expressing YFP-claudin-10b N48K showed no contact enrichment (Fig 4G). Similar results were obtained after stable expression of the two fusion proteins (Fig 4H). However, detection of the contact enrichment was more intricate in cells with stable expression when compared to the transient expression due to fragmented enrichments of the claudins at cell contacts. Hence, we used co-cultures of HEK293 cells expressing either YFP- or CFP-fusion proteins [18]. Accordingly, we quantified the enrichment of YFP and CFP that co-localized at the mixed cell contacts to discriminate between trans-interacting claudins and other potential local claudin enrichments in the plasma membrane (S4 Fig). The enrichment at contacts with co-localization of CFP and YFP was significantly lower for claudin-10b N48K than for WT (Fig 4H). Furthermore, similar differences between claudin-10b WT and claudin-10b N48K were obtained for transiently expressed fusion proteins with C-terminal GFP-tag (S5 Fig). Together, the data suggest that the p.N48K mutation does not prevent targeting of full length claudin-10b to the plasma membrane but does inhibit claudin-10b trans-interaction.

### Claudin-10b N48K promotes dimerization in cis

**Fig 5:**
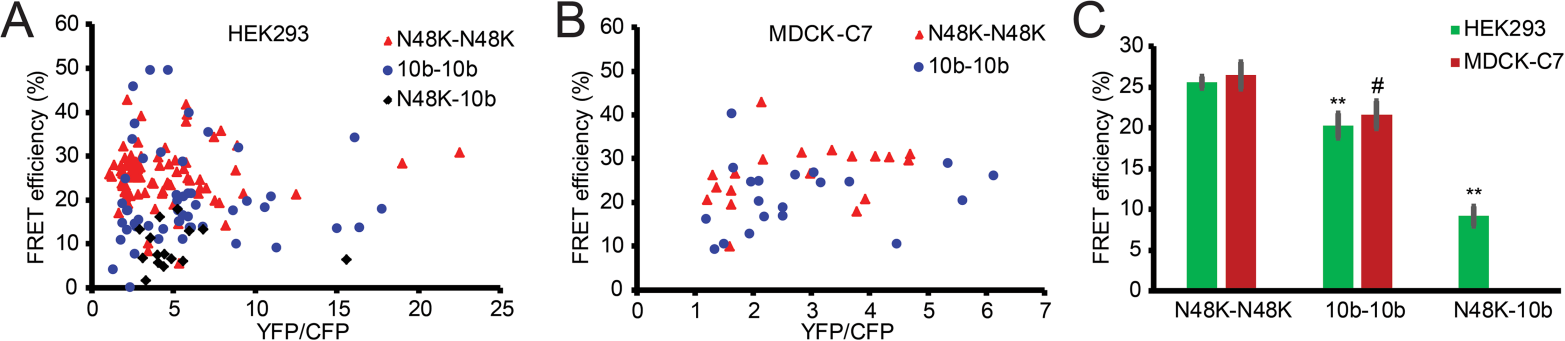
The claudin-10b N48K increases FRET efficiency in cis. (A, B) FRET efficiencies for YFP-claudin-10b WT/CFP-claudin-10b WT interaction (blue), YFP-claudin-10b N48K/CFP-claudin-10b N48K (red) and YFP-claudin-10b N48K/CFP-claudin-10b WT (black) constructs overexpressed in HEK293 cells (A) and MDCK-C7 cells (B) plotted against the YFP/CFP ratio. Only values above the critical YFP/CFP ratio as defined by Milatz et al. (2015) are shown. (C) In HEK293 (** p<0.01) and in MDCK-C7 (# p<0.05) cells average FRET efficiency is significantly higher for YFP-claudin-10b N48K/CFP-claudin-10b N48K interaction than for YFP-claudin-10b WT/CFP-claudin-10b WT interaction. Interaction between claudin-10b N48K/claudin-10b WT is significantly lower than for both claudin-10b N48K/claudin-10b N48K and claudin-10b WT/claudin-10b WT (** p<0.01, only tested in HEK cells). Statistical analysis: MDCK-C7, t-test, n = 19 (WT) and 21(N48K); HEK293, t-test + Bonferroni-Holm, n = 83 (WT), 52 (N48K) and 15 (WT-N48K). Error bars are presented with ± standard error (SEM).

Claudins assemble both in trans (between opposing membranes) and in cis (within one membrane) to form paracellular ion channels or barriers. To test whether the p.N48K mutation affects cis-oligomerization of claudin-10b we employed a fluorescence resonance energy transfer (FRET) assay on HEK293 and MDCK-C7 cells expressing CFP- and YFP-tagged claudin-10b [5, 19]. In contrast to HEK293, MDCK-C7 cells contain endogenous claudins and form TJs. In both cell types the maximum FRET efficiency was significantly higher for YFP-claudin-10b N48K/CFP-claudin-10b N48K than for YFP-claudin-10b WT/CFP-claudin-10b WT. In addition, after co-transfection in HEK293 cells, maximum FRET efficiency for both YFP- claudin-10b N48K/CFP-claudin-10b N48K and YFP-claudin-10b WT/CFP-claudin-10b WT were significantly higher than that for YFP- claudin-10b N48K /CFP-claudin-10b WT (Fig 5). Hence, the p.N48K mutation affects, but does not prevent, cis-oligomerization of claudin-10b. Together, the microscopic analysis supports that p.N48K reduces formation of claudin-10b based tight junction strands by affecting cis-interaction and by inhibition of trans-interaction.

### Homology modeling supports structural alteration of claudin-10b N48K

**Fig 6:**
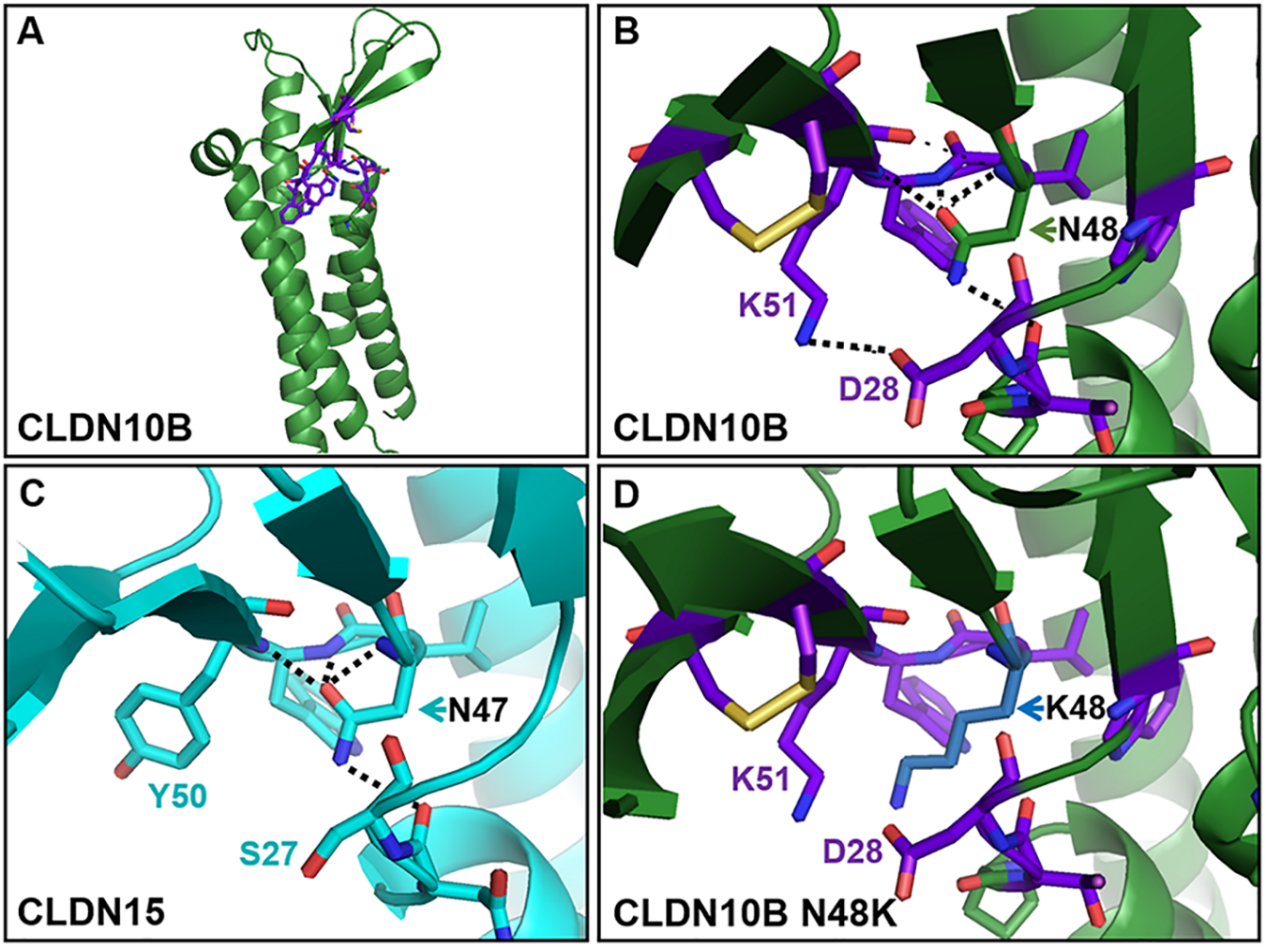
Homology modeling predicts structural alterations of claudin-10b p.N48K. (A) Homology model of the tertiary structure of claudin-10b based on crystal structure of murine claudin-15 (PBD ID: 47P9) as template. Backbone is shown as cartoon in green and residues of the claudin consensus motif (W-G/NLW-C-C), T27, D28 and K51 are shown as sticks in violet. (B) Region of claudin-10b model around N48 (green stick) in detail highlighting likely electrostatic interactions (dotted lines) between N48 and backbone of surrounding residues (T27, L49, W50) as well as potential electrostatic interaction between residues K51 and D28. (C) In the claudin-15 structure (cyan), the corresponding residue N47 participates in similar interactions (dotted lines) whereas residues Y50 (corresponding to K51) and S27 (corresponding to D28) do not interact. (D) The p.N48K substitution (blue) prevents the interactions mediated by N48 and may also disturb a D28-K51 interaction. O atoms, red; N atoms, dark blue; S yellow.

We generated a 3D homology model of claudin-10b using the crystal structure of murine claudin-15 (PBD ID: 47P9; 52% amino acid sequence identity to human claudin-10b) as template. The N48 residue is part of the consensus motif of claudins (W-G/NLW-C-C), where only claudin-15 and claudin-10b contain an asparagine (N) instead of a glycine (G). The claudin-10b model (Fig 6A) shows a fold that is very similar to the fold of the claudin-15 structure with a left-handed, four transmembrane helix bundles and a β-sheet connecting the extracellular loops (ECL) one and two [6]. Strikingly, residue N47 in the claudin-15 structure and the corresponding residue N48 in the claudin-10b model, seem to form hydrogen bonds bridging the backbone of consensus motif residues (L49 and W50 of claudin-10b) with the backbone (T27 of claudin-10b) at the transition of transmembrane helix one and ECL1 (Fig 6B and 6C). In the model, these conserved bridging interactions are disrupted by the replacement of asparagine for lysine (Fig 6D). Furthermore, a potential electrostatic interaction between residues D28 and K51 within claudin-10b could be disturbed in claudin-10b by the replacement of the uncharged for the positively charged side chain at position 48 (Fig 6B and 6D).

Together, the 3D modeling suggests that the p.N48K mutation alters the claudin-10b structure at the membrane-ECL1 transition around the claudin consensus motif. It is plausible that these intra-molecular alterations have an indirect effect on claudin-10b oligomerization. Such indirect effects of p.N48K on oligomerization are further supported by the fact that the corresponding N47 residue of claudin-15 is not part of an intermolecular interface in a polymer model reported previously [20].

## Discussion

Our work demonstrates that a homozygous missense mutation in the *CLDN10b* gene, encoding the TJ protein claudin-10b, is responsible for a phenotype characterized by generalized anhidrosis, xerostomia, alacrima and kidney damage. The missense mutation p.N48K is located in the first extracellular loop (ECL1) that distinguishes the ubiquitously expressed isoform 10b from the kidney specific isoform 10a. The loop determines the opposing electrophysiological properties of the two proteins: Isoform 10b is selective for cations and isoform 10a for anions. Notably, the N48 residue in claudin-10b is included in the consensus motif shared by all mammalian claudins.

Evidence for the pathogenic nature of the claudin-10b p.N48K was obtained by a combination of in vitro experiments. To model the effect of claudin-10b N48K on sweat production we cultured MDCK-C7 cell in matrigel to produce cysts. The expansion of cysts is driven by an osmotic gradient caused by transcellular Cl^-^ secretion and paracellular Na^+^ transport that, in a similar way, drive sweat production. However, expression of claudin-10b N48K caused a reduced lumen expansion when compared to cysts expressing claudin-10b WT. This observation suggests that the mutation reduces overall Na^+^ conductance of the cysts that may be brought about by a reduction in single cell conductance or by a reduction of the number of paracellular channels. A reduction in single channel conductance cannot be excluded although dilution and bionic potential measurements indicate that claudin-10b N48K is still able to form charge and size-selective channels. However, claudin-10b N48K showed a tendency for intracellular accumulation in MDCK cells and in sweat glands of our patients. Thus, our observations suggest that the anhidrotic phenotype is not exclusively caused by the partial loss of function mediated by junctional claudin-10b N48K but also to its reduced incorporation into TJs.

Since claudin-10b forms TJ strands and paracellular ion-channel we sought to investigate the capability of the mutated protein for TJ strand formation by ultrastructural analysis. The p.N48K substitution of claudin-10b was associated with fewer TJ strands arranged in a less complex meshwork and in contrast to claudin-10b WT, the strands formed by claudin-10b N48K consisted of separated intra-membranous particle arrays similar to those found for claudin-2, claudin-5 and claudin-3/claudin-5 and claudin-10a/claudin-10b chimeric mutants [21–23]. It has been suggested that these ultrastructural changes are related to altered claudin subtype-specific oligomerization properties [22]. The claudins interact in trans as well as in cis and we show an aberrant assembly mediated by the p.N48K substitution in cis accompanied by a marked decrease in trans-interactions at cell-cell contacts. Thus, the combined data strongly suggest that p.N48K alters both trans- and cis-interactions resulting in perturbed TJ formation. However, TJ formation is not fully prevented by the N48K mutation. Furthermore, in a 3D model based on the crystal structure of the highly homologous murine claudin-15, the N48 residue is predicted to form hydrogen bonds bridging the NLW-motif in the ECL1 with the transition between trans-membrane helix one and the ECL1. These interactions seem to be disrupted by replacing the polar asparagine for the longer and positively charged lysine. The modeling and the fact that the corresponding N47 residue of claudin-15 is not part of an intermolecular interface of a previously reported claudin-15 polymer model suggests that p.N48K indirectly affects oligomerization of claudin-10b by altering intramolecular interactions [20].

In the kidneys, an important proportion of Na^+^ reabsorption takes place within the ascending limb (TAL) of Henle’s loop through both transcellular and paracellular transport. The concerted action of apical and basolateral ion transporters generates a transepithelial voltage that drives the reabsorption of both Ca^2+^ and Mg^2+^. Within the kidney, claudin-10a is expressed exclusively in cortical proximal tubulus segments of the nephron, whereas claudin-10b is highly expressed in the medulla where 50% of the reabsorbed Na^+^ takes the paracellular route. Interestingly, ablation of the claudin-10b isoform in mouse kidney results in hypermagnesemia due to an increase in renal Mg^2+^ reabsorption. The absence of claudin-10b in the TAL results in elevated transepithelial resistance, an increased transepithelial voltage and consequently, in an increased driving force for the paracellular reabsorption of cations [15]. Additionally, absence of claudin-10b in TAL tubules resulted in increased paracellular permeability for divalent cations, possibly due to increased expression of claudin-16 and claudin-19. However, absence of claudin-10b or presence of claudin-10b N48K should not affect the claudin 16/19 pore directly since claudin-10b does not physically interact with either claudin-16 or claudin-19 [24]. The mouse model further revealed that the reduced paracellular reabsorption of Na^+^ in the TAL did not result in sodium loss. Given the observations in the mouse model and the elevated levels of Mg^2+^ in serum of our patients we hypothesized that claudin-10b p.N48K disturbs the cation permeability and in particular the paracellular Na^+^ transport. Indeed, we observed that the p.N48K mutation was associated with a reduced selectivity for Na^+^ over Cl^-^ and with a preserved transepithelial resistance in MDCK-C7 cell layers. Still, claudin-10b N48K retained the ability to interact with the hydration shell of monovalent cations Li, Na, K, Rb and Cs as judged from the higher Eisenman sequence. Furthermore, the reduction in P_Na_/P_Cl_ for the mutated protein was time dependent in culture and similar to that for clones with a weak expression of claudin-10b WT. These observations suggest that the paracellular channels formed by claudin-10b p.N48K have a subnormal Na^+^ permeability and that the number of channels is greatly reduced. In addition, the observed increased proteolytic cleavage of mutated YFP- and CFP- claudin-10b and the staining of sweat glands in our patients suggest that the N48K mutation leads to increased degradation of claudin-10b that contributes to the reduced formation of Na^+^ channels. The active transcellular transport of Cl^-^ in the sweat glands as well as in the TAL generates a transepitheleal voltage and a driving force for paracellular cation transport. In the kidney, the severely reduced Na^+^ conductivity caused by the N48K mutation is thus a likely contributing mechanism for the increased Mg^2+^ and Ca^2+^ reabsorption that results in kidney damage in our family as shown by reduced eGFR values, reduced levels of 25-hydroxy vitamin D and increased PTH levels. Furthermore, the increased reabsorption of Mg^2+^ that is associated with claudin-10b p.N48K is consistent with the kidney specific lack of claudin-10 in mice showing decreased Na^+^ permeability in the TAL accompanied by increased reabsorption of Mg^2+^. Interestingly, the increased Mg^2+^ mediated by claudin-10b p.N48K contrasts with the Mg^2+^ wasting observed in patients who carry claudin-16 or claudin-19 mutations. In combination, these findings highlight the complex renal mechanisms mediated by claudins to maintain cation homeostasis.

Taken together, our data support that claudin-10b N48K causes a disturbed relative overall permeability for cations that results in increased reabsorption of Mg^2+^ and hypermagnesemia.

In contrast to the kidney specific isoform claudin-10a, the claudin-10b isoform is ubiquitously expressed and presumably of importance for paracellular Na^+^ transport in multiple organs. In our family, affected members presented with heat intolerance due to anhidrosis, and alacrima as first symptoms in early childhood. Sweat production is mediated by IP3 acting as an intracellular messenger and the release of Ca^2+^ that opens Cl^-^ channels to the glandular lumen and thus activates transcellular Cl^-^ secretion energized by the basolateral Na^+^K^+^2Cl^-^ symporter [25]. The resulting electrochemical gradient drives paracellular Na^+^ flux that, together with AQP5 mediated water flux leads to a net secretion of a largely isotonic NaCl solution into the secretory portions of the glandular lumen [26, 27]. Our IHC analyses show claudin-10b staining in cells of the secretory portion of normal sweat glands with intense staining lining the canaliculi. This is consistent with a role for claudin-10b in the paracellular Na^+^ flux into the lumen. Compared to healthy individuals, the immunohistochemical staining of sweat glands in affected individuals showed a pronounced reduction of claudin-10b N48K in the cell peripheries and canaliculi. In sum, our data suggests that the likely mechanism behind abolished sweat production in our patients is a reduced incorporation of claudin-10b N48K into TJs mediated by altered trans- and cis-interaction properties accompanied by increased degradation of the mutated protein. Accordingly, the plausible explanation for alacrima and xerostomia is a reduced paracellular Na^+^ transport in the lacrimal and salivary glands, respectively, leading to a perturbed secretion of NaCl and water into the lumen of secretory portions [28].

In conclusion, we show that a mutation in the claudin-10b isoform results in abolished or reduced sweat production as well as a relative shift in cation resorption in the kidneys that leads to kidney damage. The affected organs contain epithelia in which transepithelial transport of NaCl is paralleled by paracellular transport of Na^+^ that is impaired by claudin-10b N48K. The combined findings expand our knowledge on the role of claudin-10b and the complex functional networks of claudins that may be useful in identifying the genetic basis for additional phenotypes caused by altered paracellular ion transport.

## Materials and methods

### Ascertainment of study subjects

The kindred examined in this study were referred to the Health Division, National Institute for Biotechnology and Genetic Engineering (NIBGE), Faisalabad, Pakistan, because of severe heat intolerance and anhidrosis. The patients were exposed to heat (45°C, 45% humidity) and perspiration was measured using starch-iodine together with healthy control individuals. It became evident that several family members also suffered from renal insufficiency. Blood and urine samples were obtained from available family members and punch skin biopsies were taken from two affected individuals. Consanguinity was ascertained over several generations and the affected individuals were related through five loops suggesting autosomal recessive inheritance (Fig 1A). The study was carried out under a protocol approved by the ethical committee of the National Institute of Biotechnology and Genetic Engineering (NIBGE), Faisalabad, Pakistan, and the Regional Ethical Committee of Uppsala, Sweden. Informed consent was obtained from all study participants or their legal guardians.

### Sequencing and sequence variant detection

SNP genotyping was performed on DNA samples from three affected family members (using the GeneChip Mapping 250K array (Affymetrix) according to the manufacturer’s protocol. Homozygosity mapping and sorting of genomic regions were performed as described previously with the dedicated software AutoSNPa [29]. Two point LOD scores were calculated for microsatellite markers using the MLINK program of LINKAGE computer package [30]. A custom enrichment design covering 6M base pairs (hg19 chr13:93278935–99228090, NimbleGen Sequence Capture Microarrays, Roche) was used to enrich for the linked region on chromosome 13. Sequencing of the enriched region was performed using the Illumina HiSeq system and variant detection was performed using v2.1 of the LifeScope Software (Life Technologies). Prediction of possible impact on protein function was performed using PolyPhen-2 analysis [31]. Variant allele frequencies were assessed using the Exome Aggregation Consortium (ExAC) database (Cambridge, MA (URL: http://exac.broadinstitute.org) accessed June 2016). Exon 1 of isoform b of the *CLDN10* gene was analyzed for the identified variant by bi-directional sequencing of genomic DNA from all available family-members using the primers: ATC AAG GAA GGA GGG CTG AG (sense) and: AGA CGC CCG TGG AGT CGG TA (antisense).

### Immunohistochemistry and immunofluorescence staining

Histological analysis of skin biopsies was performed after hematoxylin and eosin (H&E) staining. Immunofluorescence staining of claudin-1, claudin-3, claudin-10, occludin and ZO-1 α (Invitrogen, San Francisco, California rb-α-claudin-1, rb-α-claudin-3, m-α-occludin, rb-α-ZO-1, m-α-claudin-10) were added in blocking solution at a dilution of 1:100 and sections were incubated over night at 4°C. Secondary antibodies (Jackson ImmunoResearch, Newmarket, UK, Cy2 Fab gt-α-m-IgG, Cy5 Fab gt-α-rb-IgG) were applied at a dilution of 1:600 (at least 30 min at room temperature). Fluorescence images were obtained with a LSM (Zeiss LSM780, Jena, Germany).

### Transfection

HEK293 cells were transiently or stably transfected and MDCK-C7 cells were stably transfected with WT or mutated *CLDN10b* vectors containing a puromycin or neomycin resistance using PEI (Polyethylenimine, Sigma-Aldrich). Transiently transfected HEK293 cells were used for confocal laser microscopy after 24 or 48 hours. For stable transfection, MDCK-C7 cells were treated with puromycin (10 μg/ml, Sigma-Aldrich) or G418 (1000 μg/ml, Biochrom, Berlin, Germany), respectively. After 2 weeks, G418 resistant clones were picked with cloning-cylinders and the cells were further cultured with 600 μg/ml G418. Puromycin-resistant MDCK-C7 cells were pooled after 7 days, grown for further 10 days, seeded onto Millicell cell culture inserts (pore size 0.45 μm, effective area 0.6 cm^2^; Millicell-HA) and grown for further 5 days before they were mounted in an Ussing chamber. In contrast, HEK293 cells were treated with 600 U/ml G418 (Biochrom, Berlin, Germany) for 4 weeks and the resistant cells were further cultured in the presence of 150 U/ml G418.

### Immunofluorescence of three-dimensional epithelial cell culture

Cells were trypsinized to stimulate proliferation. On the following day, cells were trypsinated again and 10^4^ cells were seeded into 100 μl BD Matrigel (BD Biosciences, Heidelberg, Germany) per Lab Tek well (Lab Tek II Chambered Coverglass; Nunc). After 5 days, the cysts were fixed with 4% PFA in PBS (1 hour shaking at room temperature). Subsequently, the cysts were treated with a permeabilization solution (0.5% Triton X-100, 0.25% Saponin and 25 mM Glycine, pH 8.0, in PBS; 6 hours shaking at room temperature). For immunostaining of claudin-10, the cysts were incubated with rabbit anti-Cldn 10 (1:150, Assay bio Tech, Sunnyvale, CA, USA) overnight at 4°C. Cysts staining for diameter determination was achieved by incubation with Alexa Fluor 594 Phalloidin (1:450, Invitrogen) and DAPI (1:500, Roche) overnight at 4°C. All cysts were washed with permeabilization solution (6 hours shaking at room temperature) and the solution was changed every half hour. Cysts intended for diameter determination were subsequently covered with ProTaqs Mount Fluor (Biocyc, Luckenwalde, Germany). Dyed Cldn10-cysts were further incubated with Phalloidin-Dy-647P1 (1:1000, Dyomics GmbH, Jena, Germany), anti-rabbit IgG Alexa Fluor 488 (1:400, Molecular Probes), and DAPI (1:500) overnight at 4°C. Dyed Cldn10-cysts were washed with permeabilization solution (6 hours shaking at room temperature; solution changed every half hour). The cysts were subsequently covered with ProTaqs Mount Fluor. Fluorescence images were obtained with a LSM (Zeiss LSM780, Jena, Germany).

### Cyst diameter evaluation

Z-stack images of cysts stained with DAPI and phalloidin were recorded by confocal laser scanning microscopy (Zeiss LSM780, x20). Within each stack, the layer with the largest diameter of each individual cyst was identified, the diameter marked with a straight line and the length of each line determined, using the Zeiss ZEN software. Mean diameters of each stack were calculated and averaged to obtain mean ± SEM for each cell clone. For size distribution histograms, individual cyst diameters were sorted into size intervals (width 15 μm), and the relative frequency was calculated by the ratio of cyst number per interval divided by total number of cysts of each cell clone. For visualization of the shift in the distribution, normal distributions were calculated for each histogram.

### Freeze fracture electron microscopy

For freeze fracturing, HEK293 cells were washed twice with PBS with MgCl_2_ and CaCl_2_ (Sigma-Aldrich), fixed with phosphate-buffered 2.5% glutaraldehyde (Sigma-Aldrich) for 2 hours at room temperature. Cells were washed with PBS and stored in 0.1% glutaraldehyde in PBS at 4°C. Electron microscopy was performed similar as described before [32].

### Live cell imaging for trans-interaction analysis

For claudin trans-interaction analysis, HEK293 cells, a cell line devoid of TJs, was used. As a measure for claudin-10b trans-interaction, enrichment of the transfected YFP-/CFP-claudin-10b constructs at contacts between two claudin-expressing cells was analyzed similar as described previously [5, 22]. Briefly, one or two days after transient transfection or replating stable lines, cells were transferred to Hanks' Balanced Salt Solution (HBSS) pH 7.4 with Ca^2+^, Mg^2+^, glucose, sodium bicarbonate, without phenol red (Thermo Scientific) and examined with a LSM 780 system (Carl Zeiss, Jena, Germany). Randomly chosen cells were analyzed using the ZEN software (Carl Zeiss, Jena, Germany) and YFP intensity profiles of confocal images. The enrichment factor (EF) was calculated as the intensity of the YFP signal at contact between two claudin-expressing cells divided by the sum of the intensities at contact between these two claudin-expressing cells and neighboring non-expressing cells. EF >1 indicates enrichment [5].

### FRET assay

For analysis of the cis-interaction between claudin-10b constructs along the plasma membrane of one cell, FRET analysis was performed. HEK293 were co-transfected with two plasmids encoding a CFP-claudin-10b (mutated or WT) and an YFP-claudin-10b (mutated or WT) fusion protein, respectively and analyzed at cell-cell contacts as described previously [19]. Before and after acceptor bleaching, CFP and YFP intensity was detected. Since the FRET efficiency EF depends on the acceptor/donor ratio, EF was plotted as a function of YFP/CFP for each acceptor/donor pair [33, 34]. Signals were calibrated using an YFP-CFP tandem protein, so that equal YFP and CFP intensities denote equal amounts of these proteins. Curve fitting and data analysis to obtain the average FRET efficiency were carried out as described previously [19].

### SDS-PAGE and western blot

HEK293 cells were washed with ice cold PBS (with Ca^2+^ and Mg^2+^), lysed on ice with 1% (v/v) Trition X-100 in PBS containing EDTA-free protease inhibitor cocktail (Roche, Mannheim, Germany) and centrifuged at 10.000 x g (5 min, 4°C). Supernatants were mixed with Laemmli buffer, boiled and loaded on 10% SDS gels, transferred on PVDF membranes. The CFP/YFP-fusion proteins were detected using mouse anti-GFP (JL-8, Takara Clontech, Saint-Germain-en-Laye, France) and HRP-coupled (Jackson Immunoresearch) anti-mouse antibodies.

### Homology modeling

The protomer model of human claudin-10b was generated using the crystal structure of murine claudin-15 (PDB ID: 4P79) as template and Swissmodel (http://swissmodel.expasy.org) [6, 20, 35, 36]. Model quality was estimated employing the QMEAN server (http://swissmodel.expasy.org/qmean) [37]. In addition, Modeller was used and resulted in a similar model [38]. Images of the structures and models were generated using PyMOL (version 1.5.0.4 Schrödinger, LLC).

### Dilution potential measurements

Cell culture inserts were mounted into Ussing chambers and the water-jacketed gas lifts on both sides were filled with 10 ml of a bath solution containing 119 mM NaCl, 21 mM NaHCO_3_, 5.4 mM KCl, 1.2 mM CaCl_2_, 1 mM MgSO_4_, 3 mM HEPES, and 10 mM D(+)-glucose. The solution was constantly bubbled with 95% O_2_ and 5% CO_2_, to ensure a pH value of 7.4 at 37°C. After equilibration, 5 ml of the apical or basolateral solution were replaced by a bath solution which, instead of 119 mM NaCl, contained 238 mM mannitol. The resulting voltage step (`dilution potential') was converted into the permeability ratio, P_Na_/P_Cl_, as described previously [14]. Eisenman sequences were determined analogously by using solutions containing 119 mM XCl (LiCl, KCl, RbCl, or CsCl, respectively), instead of NaCl. P_X_/P_Na_ was calculated from the resulting ‘bi-ionic potentials’ and the P_Na_/P_Cl_ obtaind from the dilution potential measurements. Complete equations are described by Günzel et al [14].

## Supporting information

S1 Table Pulmonary function of two adult family members who are homozygous for the claudin-10b N48K variant. Spirometry revealed normal lung function in both individuals. FVC: Forced Vital Capacity, FEV1: Forced expiratory volume in 1 second, FEV1%: FEV1/FVC ratio. Predicted: Predicted normal values, %Predicted: Patient values of predicted values.
(DOCX)

S1 Fig Eccrine sweating estimated by iod-starch test. Iod and starch were applied to different body parts of a healthy individual (left) who was age/gender matched with an affected individual (right) followed by exposure to heat (45°C). Sweat secretion causes a dark blue color change in the healthy subject (left) whereas reduced or absent sweating results in faint or no color as shown in the affected individual (right).
(TIF)

S2 Fig Immunofluorescence co-staining of claudin-10b and other TJ proteins in normal sweat glands. Claudin-10 **(**cldn10) shows strong staining of membranes facing the canaliculi as well as the gland lumen (A-C) whereas neither claudin-1 (cldn1) nor claudin-3 (cldn3) stain membranes facing the canaliculi (A and B; see exclusively green canalicular staining in the merged figures in A and B) (arrows). Claudin-1 and claudin-10 show strong extra-junctional staining (A) which could represent basal infoldings. Furthermore, claudin-1 is detected in all cells of the sweat gland coil whereas claudin-10 is not (see merge figure A). Like claudin-10, occludin (Occl) and ZO-1 show strong staining of membranes facing the canaliculi as well as adjacent to the glandular lumen (C, D) but, in contrast to claudin-10b, no basal staining is observed. Scale bars: 20μm; arrows, canaliculi; arrow heads, basal staining.
(TIF)

S3 Fig The N48K mutation enhances degradation of claudin-10b in HEK293 cells. Live cell imaging of co-culture of stable HEK293 lines expressing (A) CFP- (green) or YFP-claudin-10b WT (red) and (B) CFP- (green) or YFP-claudin-10b N48K (red). Cytosolic fluorescence was found much more frequent for cells expressing claudin-10b N48K when compared to cells expressing claudin-10b WT, indicating CFP/YFP cleavage of the fusion protein. Living HEK293 cells were imaged by laser scanning microscopy (LSM). Plasma membrane of cells was labeled with CellmaskTM (blue) and imaged together with the CFP and YFP. Bar 20 μm. (C) Western Blot analysis after SDS-PAGE of cell lysates of stable HEK293 lines expressing claudin-10b WT (10Bwt) or claudin-10b N48K (10BN48K). For all constructs, beside a band corresponding to the full length CFP/YFP-claudin-10b fusion protein (~50 kDa, upper arrow) cleavage products (lower arrows) were found (representative blot). Degradation products were more prominent for CLDN10b p.N48K. (D) Densitometric quantification of the ratio between full length to degradation bands revealed a significantly lower ratio in cultures expressing claudin-10b N48Kwhen compared to cultures expressing claudin-10b WT. CFP- and YFP- fusions were grouped, n = 8–12, * = p<0.01, unpaired t-test). Error bars are presented with ± standard error (SEM).
(TIF)

S4 Fig Claudin-10b trans-interaction of WT constructs. Co-culture of HEK293 cells with a stable expression of (A) CFP-claudin-10b WT (green), (B) YFP-claudin-10b WT (red) and (C) merged. Enrichment of claudin-10b WT at contacts between two cells expressing CFP-claudin-10b WT (green arrows), YFP-claudin-10b WT (red arrows) and CFP-claudin-10b WT or YFP-claudin-10b WT (white arrows, yellow overlay) indicates claudin trans-interactions. (D and E) Magnified images of white boxes in (C). (F) Plasma membrane of cells was labeled with Cellmask^TM^ (blue) and imaged together with the CFP and YFP. Living HEK293 cells were imaged by laser scanning microscopy (LSM).
(TIF)

S5 Fig Inhibition of trans-interactions and perturbed formation of tight junction strand for C-terminallly tagged claudin-10b-N48K-GFP. (A, B) LSM analysis of subcellular distribution of claudin-10b constructs with C-terminal AcGFP-tag (GFP-variant). (A) Similar as for YFP-claudin-10b-wt (N-terminal GFP-variant, Fig 2E) claudin-10b-wt-AcGFP was often enriched at contacts between claudin-expressing cell (arrows) but showed only weak signals in other areas of the plasma membrane (arrowheads). (B) In contrast, claudin-10b-N48K-AcGFP showed no contact enrichment (arrows) but more uniform distribution through out the plasma membrane (arrowheads), also similar to YFP-claudin-10b-N48K (Fig 2F). In general the C-terminally tagged constructs (A, B) showed more intracellular signals than the N-terminally tagged constructs (Fig 2E and 2F). Bar, 5 μm. (C-D) Freeze fracture electron microscopy of HEK293 cells transiently expressing Claudin-10b-AcGFP constructs. (C) Similar as for YFP-claudin-10b WT (Fig 2C), for claudin-10b WT-AcGFP complex meshworks of branched continuous-type tight junction strands were detected on the protoplasmic face (P-face, PF) and as mainly particle-free grooves (arrowhead) on the exoplasmic face (E-face, EF) of the plasma membrane. (D) In contrast, but similar to YFP-claudin-10b N48K, for claudin-10b N48K AcGFP only few tight junction strands and less complex meshworks were detected on the P-face as rows of rather separated intramembranous particles (white arrow). Bar, 200 nm.
(TIF)
