# Supplementary material for: Altered paracellular cation permeability due to a rare CLDN10B variant causes anhidrosis and kidney damage
Source: PLoS Genet. 2017 Jul 7;13(7):e1006897. doi: 10.1371/journal.pgen.1006897 (PMC5521874; doi:10.1371/journal.pgen.1006897)
Supplement: S1 Table — Spirometry revealed normal lung function in both individuals. FVC: Forced Vital Capacity, FEV1: Forced expiratory volume in 1 second, FEV1%: FEV1/FVC ratio. Predicted: Predicted normal values, %Predicted: Patient values of predicted values. (DOCX) [file pgen.1006897.s001.docx]

|  | **Individual 14** (32 years) | | | **Individual 16** (24 years) | | |
| --- | --- | --- | --- | --- | --- | --- |
|  | Measured | Predicted | % Predicted | Measured | Predicted | % Predicted |
| **FVC** | 2.86 | 2.79 | 102 | 4.93 | 3.48 | 142 |
| **FEV1** | 2.45 | 2.43 | 101 | 3.08 | 3.00 | 103 |
| **FEV1%** | 0.86 | 0.84 | 102 | 0.63 | 0.82 | 77 |
