# Supplementary figures and images for: Altered paracellular cation permeability due to a rare CLDN10B variant causes anhidrosis and kidney damage
Source: PLoS Genet. 2017 Jul 7;13(7):e1006897. doi: 10.1371/journal.pgen.1006897 (PMC5521874; doi:10.1371/journal.pgen.1006897)

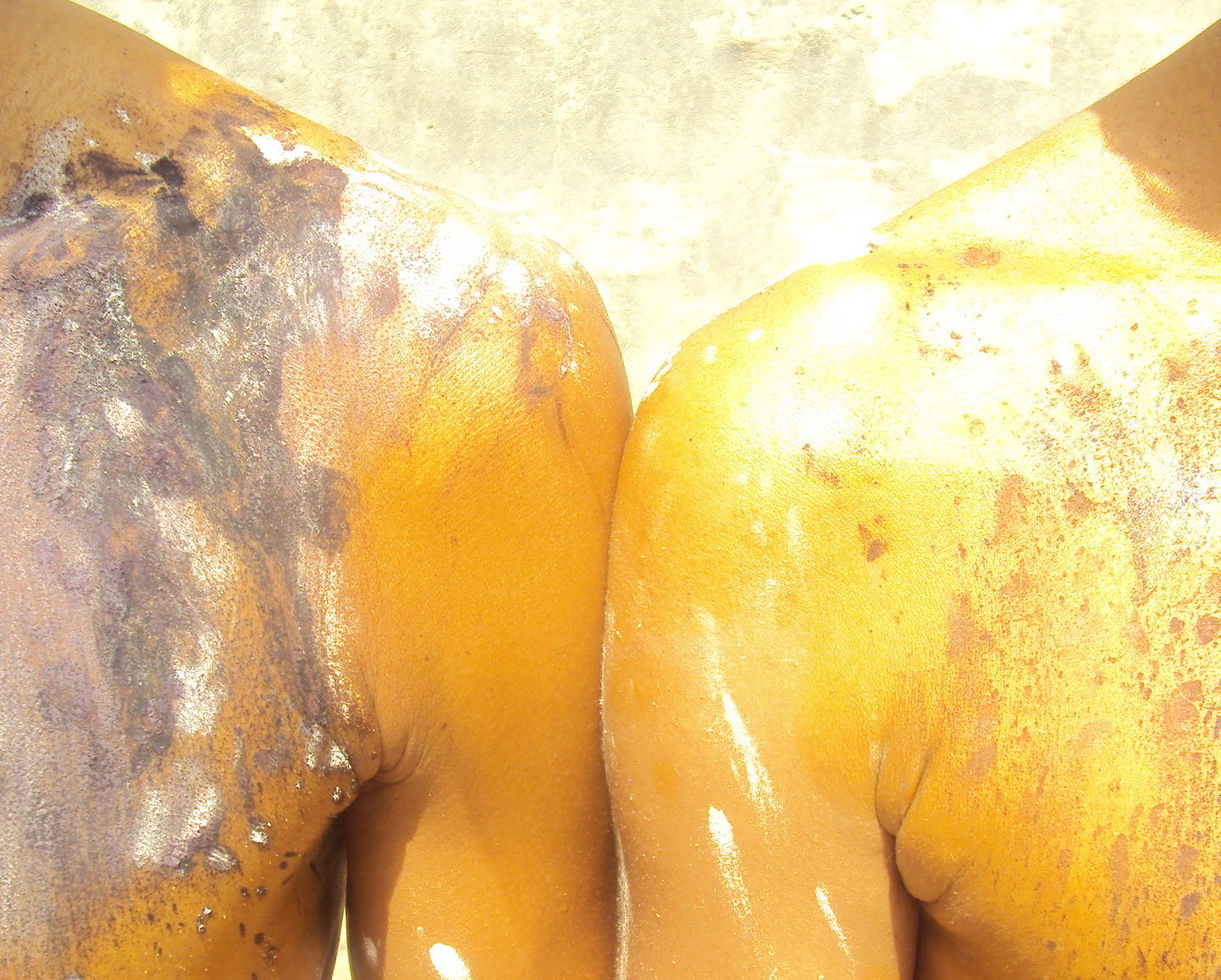

Supplement: S1 Fig — Iod and starch were applied to different body parts of a healthy individual (left) who was age/gender matched with an affected individual (right) followed by exposure to heat (45°C). Sweat secretion causes a dark blue color change in the healthy subject (left) whereas reduced or absent sweating results in faint or no color as shown in the affected individual (right). (TIF) [file pgen.1006897.s002.tif]

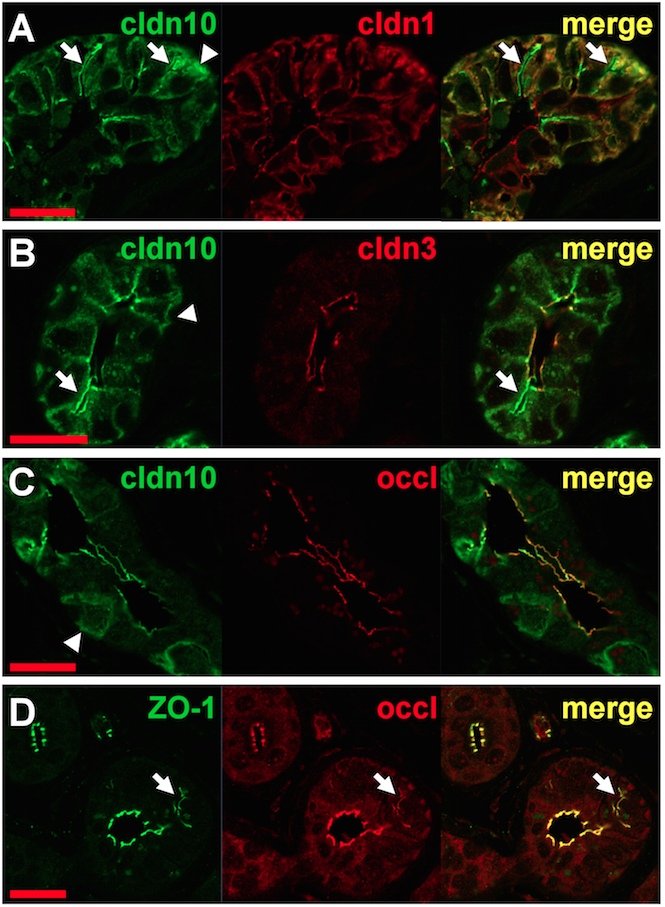

Supplement: S2 Fig — Claudin-10 (cldn10) shows strong staining of membranes facing the canaliculi as well as the gland lumen (A-C) whereas neither claudin-1 (cldn1) nor claudin-3 (cldn3) stain membranes facing the canaliculi (A and B; see exclusively green canalicular staining in the merged figures in A and B) (arrows). Claudin-1 and claudin-10 show strong extra-junctional staining (A) which could represent basal infoldings. Furthermore, claudin-1 is detected in all cells of the sweat gland coil whereas claudin-10 is not (see merge figure A). Like claudin-10, occludin (Occl) and ZO-1 show strong staining of membranes facing the canaliculi as well as adjacent to the glandular lumen (C, D) but, in contrast to claudin-10b, no basal staining is observed. Scale bars: 20μm; arrows, canaliculi; arrow heads, basal staining. (TIF) [file pgen.1006897.s003.tif]

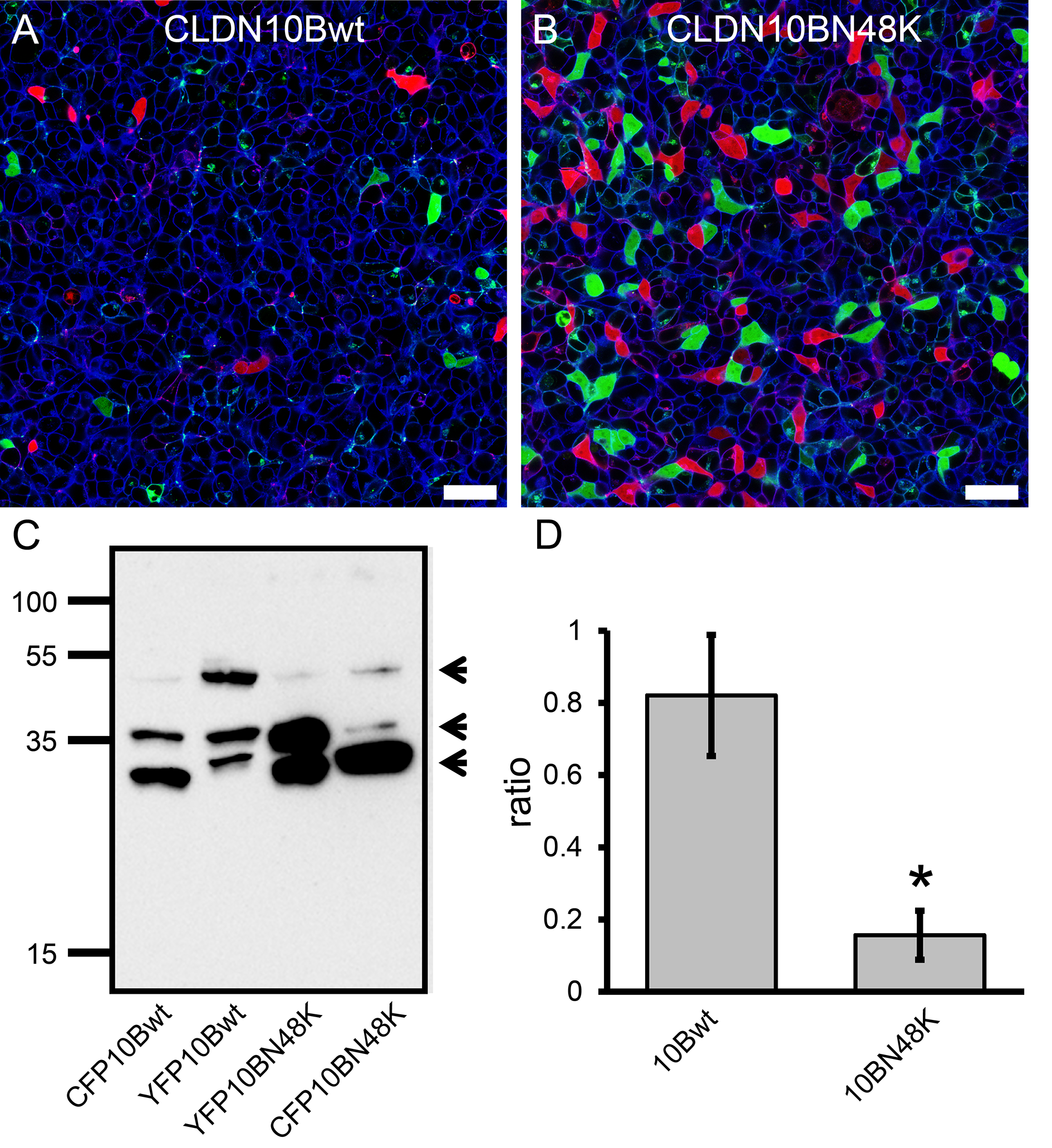

Supplement: S3 Fig — Live cell imaging of co-culture of stable HEK293 lines expressing (A) CFP- (green) or YFP-claudin-10b WT (red) and (B) CFP- (green) or YFP-claudin-10b N48K (red). Cytosolic fluorescence was found much more frequent for cells expressing claudin-10b N48K when compared to cells expressing claudin-10b WT, indicating CFP/YFP cleavage of the fusion protein. Living HEK293 cells were imaged by laser scanning microscopy (LSM). Plasma membrane of cells was labeled with CellmaskTM (blue) and imaged together with the CFP and YFP. Bar 20 μm. (C) Western Blot analysis after SDS-PAGE of cell lysates of stable HEK293 lines expressing claudin-10b WT (10Bwt) or claudin-10b N48K (10BN48K). For all constructs, beside a band corresponding to the full length CFP/YFP-claudin-10b fusion protein (~50 kDa, upper arrow) cleavage products (lower arrows) were found (representative blot). Degradation products were more prominent for CLDN10b p.N48K. (D) Densitometric quantification of the ratio between full length to degradation bands revealed a significantly lower ratio in cultures expressing claudin-10b N48Kwhen compared to cultures expressing claudin-10b WT. CFP- and YFP- fusions were grouped, n = 8–12, * = p<0.01, unpaired t-test). Error bars are presented with ± standard error (SEM). (TIF) [file pgen.1006897.s004.tif]

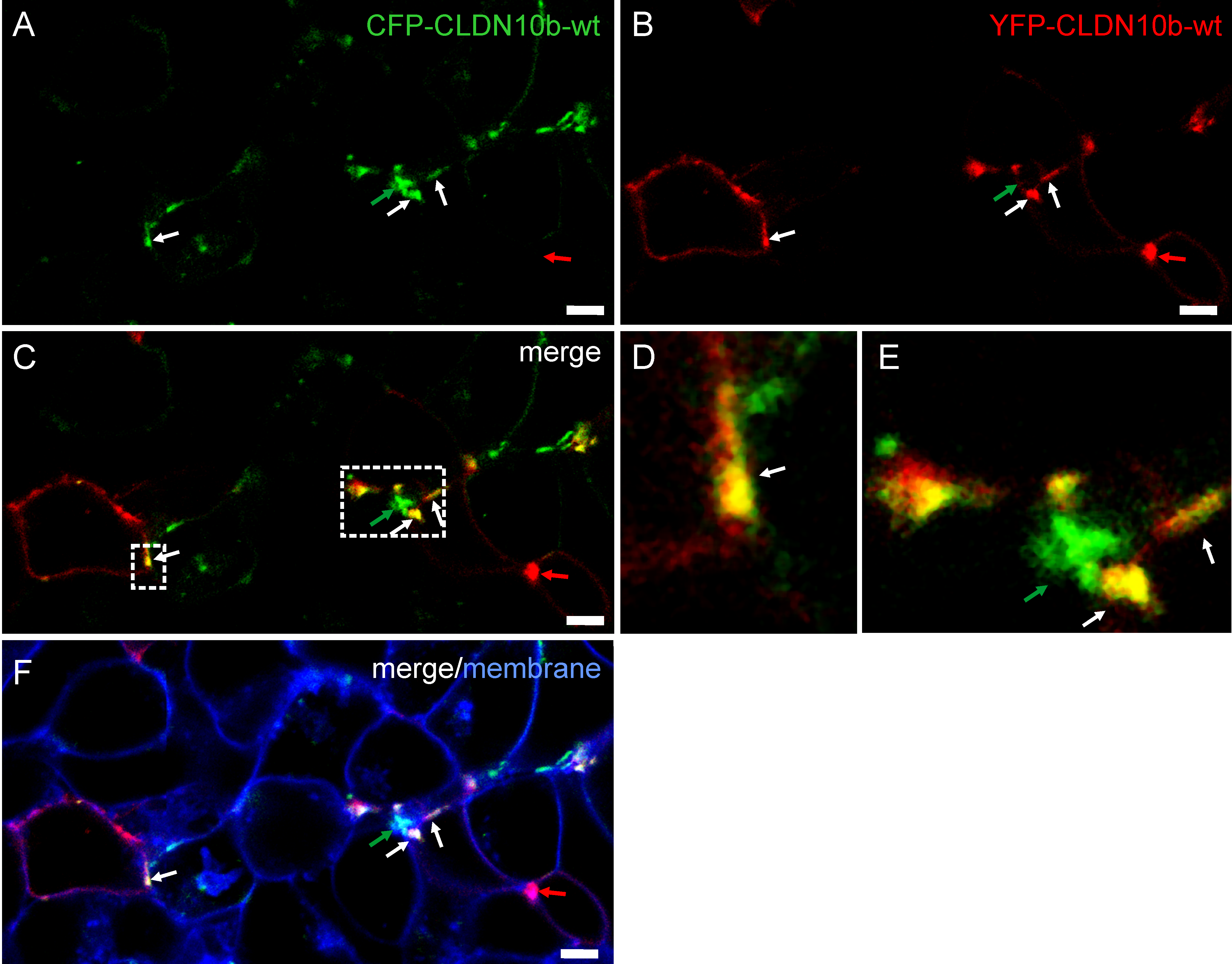

Supplement: S4 Fig — Co-culture of HEK293 cells with a stable expression of (A) CFP-claudin-10b WT (green), (B) YFP-claudin-10b WT (red) and (C) merged. Enrichment of claudin-10b WT at contacts between two cells expressing CFP-claudin-10b WT (green arrows), YFP-claudin-10b WT (red arrows) and CFP-claudin-10b WT or YFP-claudin-10b WT (white arrows, yellow overlay) indicates claudin trans-interactions. (D and E) Magnified images of white boxes in (C). (F) Plasma membrane of cells was labeled with CellmaskTM (blue) and imaged together with the CFP and YFP. Living HEK293 cells were imaged by laser scanning microscopy (LSM). (TIF) [file pgen.1006897.s005.tif]

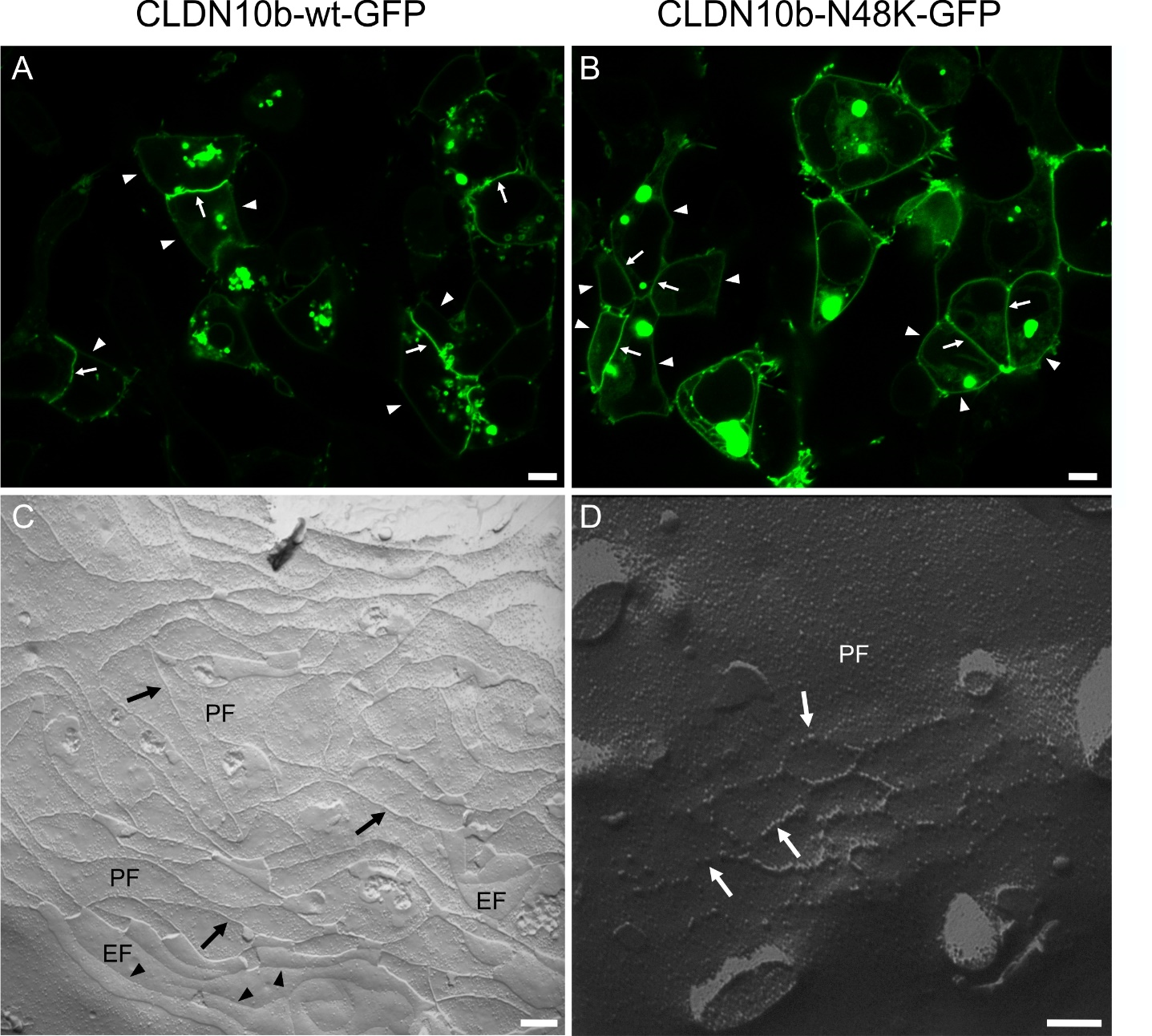

Supplement: S5 Fig — (A, B) LSM analysis of subcellular distribution of claudin-10b constructs with C-terminal AcGFP-tag (GFP-variant). (A) Similar as for YFP-claudin-10b-wt (N-terminal GFP-variant, Fig 2E) claudin-10b-wt-AcGFP was often enriched at contacts between claudin-expressing cell (arrows) but showed only weak signals in other areas of the plasma membrane (arrowheads). (B) In contrast, claudin-10b-N48K-AcGFP showed no contact enrichment (arrows) but more uniform distribution through out the plasma membrane (arrowheads), also similar to YFP-claudin-10b-N48K (Fig 2F). In general the C-terminally tagged constructs (A, B) showed more intracellular signals than the N-terminally tagged constructs (Fig 2E and 2F). Bar, 5 μm. (C-D) Freeze fracture electron microscopy of HEK293 cells transiently expressing Claudin-10b-AcGFP constructs. (C) Similar as for YFP-claudin-10b WT (Fig 2C), for claudin-10b WT-AcGFP complex meshworks of branched continuous-type tight junction strands were detected on the protoplasmic face (P-face, PF) and as mainly particle-free grooves (arrowhead) on the exoplasmic face (E-face, EF) of the plasma membrane. (D) In contrast, but similar to YFP-claudin-10b N48K, for claudin-10b N48K AcGFP only few tight junction strands and less complex meshworks were detected on the P-face as rows of rather separated intramembranous particles (white arrow). Bar, 200 nm. (TIF) [file pgen.1006897.s006.tif]
